# Disparities in reported psychosocial assessment across public and private maternity settings: a national survey of women in Australia

**DOI:** 10.1186/1471-2458-13-632

**Published:** 2013-07-04

**Authors:** Nicole Reilly, Sheree Harris, Deborah Loxton, Catherine Chojenta, Peta Forder, Jeannette Milgrom, Marie-Paule Austin

**Affiliations:** 1 Perinatal and Women’s Mental Health Unit, St John of God Health Care and University of New South Wales, PO Box 261, Burwood, NSW 1805, Australia; 2 Australian Longitudinal Study on Women’s Health, University Drive, Callaghan, NSW 2308, Australia; 3 Research Centre for Gender, Health & Ageing, University of Newcastle, University Drive, Callaghan, NSW 2308, Australia; 4 Melbourne School of Psychological Sciences, University of Melbourne, Melbourne, Victoria 3010, Australia; 5 Department of Clinical and Health Psychology and Parent-Infant Research Institute, Austin Health, Heidelberg Repatriation Hospital, Heidelberg Heights, Victoria 3081, Australia

**Keywords:** Antenatal, Postnatal, Depression screening, Psychosocial assessment, Health equity

## Abstract

**Background:**

Psychosocial assessment and depression screening is now recommended for all women who are pregnant or have recently given birth in Australia. Existing studies which have examined the extent of participation by women in such population-based programs have been primarily concerned with depression screening rather than a more comprehensive examination of psychosocial assessment, and have not been sufficiently inclusive of the 30% of women whose maternity care is provided in the private sector. Whether there are disparities in equity of access to perinatal psychosocial assessment is also unknown.

**Methods:**

A sub-sample of women (N = 1804) drawn from the Australian Longitudinal Study on Women’s Health participated in the study. Overall rates of assessment across five psychosocial domains (current emotional health; mental health history; current level of support; current drug or alcohol use; experience of domestic violence or abuse), as well as receipt of mental health promotion information, were examined. Log binomial regression was performed to investigate whether there were socio-demographic or health system inequalities among women who are and are not assessed across each domain.

**Results:**

Two-thirds of women (66.8%) reported being asked about their current emotional health in the antenatal period, increasing to 75.6% of women in the postnatal period. Rates decreased markedly for reported assessment of mental health history (52.9% during pregnancy and 41.2% postnatally). Women were *least* likely to be asked about their experience of domestic violence or abuse in both the antenatal and postnatal periods (in total, 35.7% and 31.8%, respectively).

In terms of equity of access to psychosocial assessment, women who gave birth in the public hospital sector were more likely to report being assessed across all domains of assessment in the antenatal period, compared with women who gave birth in the private sector, after adjusting for other significant covariates. State of residence was associated with reported rates of assessment across all domains in both the antenatal and postnatal periods. Women from non-English speaking backgrounds and women with more than one child were less likely to be assessed across various domains.

**Conclusion:**

This study provides an important insight into the reported overall penetration of and access to perinatal psychosocial assessment among a sample of women in Australia. Opportunities to minimise the current shortfall in assessment rates, particularly in the private sector, and for ongoing monitoring of assessment activity at a national level are discussed.

## Background

There have been a number of important developments in perinatal mental health research, policy and clinical practice in Australia over the last decade. Of particular national significance are the National Postnatal Depression Program [1], development of the National Action Plan for Perinatal Mental Health [2], establishment of the National Perinatal Depression Initiative (NPDI) [3], and introduction of the NHMRC-endorsed *beyondblue* Clinical Practice Guidelines for Depression and Related Disorders in the Perinatal Period [4]. These initiatives endorse universal, routine psychosocial assessment of all women as a key component of pregnancy and postnatal care.

Psychosocial assessment in the perinatal period refers to the clinical evaluation of a broad number of psychosocial risk factors that may contribute to the mental health outcomes of a woman and her infant [5]. The inclusion of relevant screening tools (e.g., the Edinburgh Postnatal Depression Scale (EPDS)[6] may facilitate the identification of current distress and depressive symptoms. The NPDI includes provision for the EPDS to be used for depression screening during pregnancy and the postnatal period, and its administration across the perinatal period is recommended in the 2011 Guidelines. The 2011 Guidelines also suggest, as a good practice point, that all women be asked about their mental health history, level of support, drug and alcohol use, and past or current experience of abuse, and that psycho-education (including mental health promotion information) be routinely provided in the perinatal period [4]. These recommendations and good practice points are complemented by a number of state-based initiatives developed prior to or in tandem with the 2011 Guidelines (e.g., [7,8]), and are grounded in a health promotion, prevention and early intervention framework.

In principle, the population-based approach of Australia’s perinatal mental health initiatives provides an opportunity for routine psychosocial assessment to be available to all women who are pregnant or have recently given birth. However, the implementation and delivery of psychosocial assessment is made more complex by the context in which maternity care is delivered in Australia, and its related mix of Commonwealth, State/Territory and private funding. More than half of pregnant women (55%) receive antenatal care in the public sector, with the remainder of women receiving care from private obstetricians (30%) or General Practitioners (15%) [9]. Public hospital maternity care is provided at no cost to women, whereas private maternity services, although subsidised through Medicare Benefits Schedule and private health insurance rebates, require varying out-of pocket patient contributions for particular services [10]. The private health insurance premiums paid for by this group of women will also vary according to the type and level of premium purchased, the size of the family purchasing insurance, and the particular fund of which she is a member. In this context, private health insurance is an indicator of socioeconomic advantage in Australia, as it is only accessible to people with sufficient financial resources to purchase it.

Postnatally, the provision of care in the private sector does not extend beyond delivery and the immediate postpartum period. Rather, postnatal services are provided by States/Territories and are fee free and accessible to *all* women, although there is significant variation across jurisdictions in the level of services provided. Continuity of care between Australia’s hospital maternity care system and the post-birth community-based primary care system has been identified as a particularly important element of maternity care, yet is difficult to achieve for many women within the context of the current funding structures, noted workforce shortages and service capabilities [11]. In addition, access to quality maternity services is poorer for women living in rural and remote areas and for Indigenous women [10].

Reporting on priority health areas requires an evolving evidence base to monitor the level of participation in key initiatives, and to inform decision making and quality improvement. Despite the increased investment in perinatal mental health in Australia, only a small number of studies have examined the extent of participation in our psychosocial assessment programs. A small 2004 survey of 66 Victorian hospitals conducted indicated that only half of those surveyed used a psychosocial assessment tool during the postnatal stay, with significant variation in the content of this assessment. This variation included greater attention paid to emotional wellness than to social issues such as violence or substance misuse, and an apparent reliance on antenatal, rather than postnatal, assessment [12]. A 2011 survey of 14 maternity hospitals also showed a greater emphasis on assessment of antenatal rather than postnatal emotional health [13]. Neither of these studies included private maternity hospitals, although a broad mapping of perinatal mental health activity in Australia showed that routine psychosocial assessment is less likely to be provided in the private sector [14].

Other Australian studies have been more exclusively concerned with uptake of depression screening in the perinatal period [15-17]. Overall participation rates in these studies range from 69.1% [17] to 85.7% [13], although differences in sample size, study location (e.g., urban vs. rural), study period (e.g., 2002 vs. 2011), and methodology (e.g., hospital survey vs. medical record review) should be noted. A 2010 self-report survey of nearly 29,000 mothers across Australia suggests much lower rates of participation at a national level, with only 37% and 53% of mothers reporting that they completed a questionnaire about whether they were experiencing depression during pregnancy and/or in the first postnatal year, respectively [18].

While these reports provide some insight into variations in the overall uptake of psychosocial assessment (particularly depression screening), no studies have explicitly examined the penetration of a more comprehensive approach to assessment in Australia nor the characteristics of women who have and have not been assessed. Consequently, whether there are disparities in equity of access to perinatal psychosocial assessment is unknown. This is of particular interest given that studies in other areas of women’s health have reported socio-demographic inequalities among women who do and do not participate in population-based screening programs in Australia, including lower rates of cervical screening among socio-economically disadvantaged women [19], and lower rates of first and second trimester ultrasound and maternal serum screening among Aboriginal women and women who are younger, who live in more remote areas, or who are more socio-economically disadvantaged [20]. Identification of the characteristics of women who have not experienced psychosocial assessment during the perinatal period will directly inform strategies to minimise the current shortfall in assessment rates.

Using data from a sample of women who were pregnant or who had recently given birth in Australia, the aims of this study were twofold: first, to examine the proportion of women who were assessed for a range of psychosocial risk factors in the antenatal and postnatal periods and second, to investigate whether there are disparities in equity of access to assessment at socio-demographic or health service levels. Our large cohort provided an ideal opportunity to investigate receipt of perinatal psychosocial assessment by partner status, parity, socioeconomic indicators, area of residence, background language and maternity care sector.

## Methods

### Setting and data source

This study utilises data collected from the Australian Longitudinal Study on Women’s Health (ALSWH). ALSWH is a population-based study that began in 1996, with over 40,000 participants across Australia. The participants were randomly sampled from the Medicare database which includes all permanent residents of Australia, with over-sampling in rural and remote areas. Further details of the recruitment methods have been described elsewhere [21,22]. Women participating in ALSWH and complete mailed surveys on such topics as physical and mental health, health behaviours, health service use, and other socio-demographic measures. The project is jointly conducted by researchers at the University of Newcastle and the University of Queensland, and it is funded by the Australian Government Department of Health and Ageing.

This research was a sub-study of the ALSWH, and thus involved the completion of an additional survey by a sub-group of women from the cohort born between 1973 and 1978. These women were broadly representative of the population of Australian women in this age group, with some overrepresentation of tertiary educated women and women from English speaking backgrounds [23]. These women have been surveyed five times over a 13-year period (in 1996, 2000, 2003, 2006 and 2009). The survey instrument for the current sub-study was developed by the investigators and pilot tested for comprehensibility. The final survey was approved by the ALSWH Publications, Substudies and Analyses Committee and comprised 81 fixed-choice and four opened-ended questions about pregnancy and postnatal care, emotional health and wellbeing, labour and delivery, breastfeeding, health service use and socio-demographic factors. Protocols and policies for conducting sub-studies through ALSWH are well documented elsewhere (e.g., [24,25].

### Participants and recruitment

The sampling frame for the current ALSWH sub-study was restricted to women who: i) had responded to ALSWH Young Survey 5 in 2009 (N = 8200); ii) had given birth to a child during or after July 2007 or who were in their second or third trimester of pregnancy at Survey 5 (N = 2397). These study years took into consideration sample size and power estimates and timing of survey completion relative to key national initiatives, and ensured participant recall was limited to a period of four years or less.

Primiparous women who were in their first trimester of pregnancy, or who were unsure of their pregnancy status, at the time of the Survey 5 (N = 41) were not included in the sampling frame, to minimise the likelihood that women who had not recently had a child be sent the survey.

**Figure 1:**
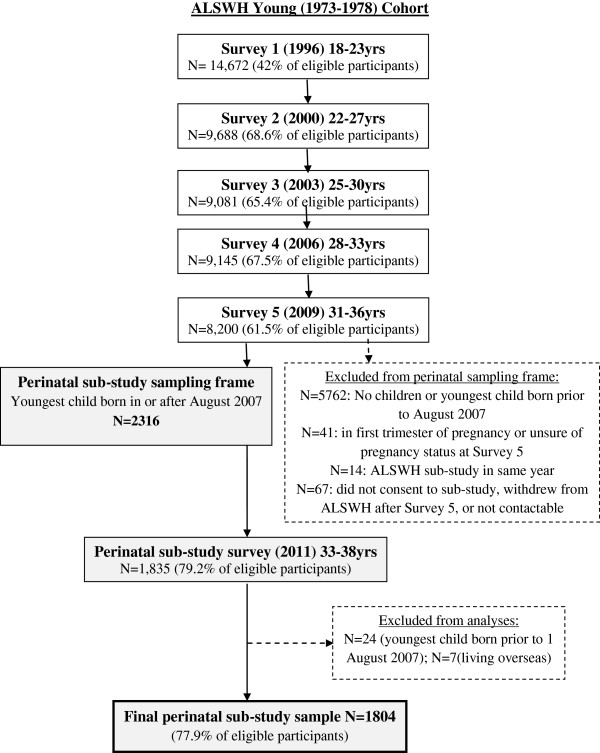
Pathway to study sample selection.

Fourteen women (0.5%) who were participating in a separate ASLWH sub-study in the same year were subsequently excluded from the sample, as were 67 women (2.8%) who had requested to not be included in further sub-studies, did not have current mailing details or had withdrawn from ALSWH after completing Survey 5. Thus the final study sampling frame comprised 2316 women. The pathway to sample selection, and recruitment and retention fractions for the cohort from which this sub-sample was drawn, are summarised in Figure 1.

### Procedure

Mailed questionnaires were used to collect data about pregnancy and postnatal health, psychosocial assessment, health care and socio-demographic factors. Respondents were invited to answer survey questions in relation to their youngest child, and the pregnancy for that child (henceforth referred to as the index child). All index children were born between July 2007 and June 2011 inclusive. Written informed consent was obtained from all participants for the collection of study data and the de-identified publication of results.

### Measures

#### Perinatal mental health information

Respondents were asked if they were “given any information about emotional well-being during pregnancy and early parenthood (e.g., about depression, anxiety, parenting stress)”, with yes/no response options for both the antenatal and postnatal periods.

#### Domains of perinatal psychosocial assessment

Respondents were asked if they had been asked by their health practitioner/s (general practitioner; obstetrician; midwife; other) about five domains of psychosocial health during pregnancy and/or the postnatal period (up to 12 months postpartum): i) current emotional health; ii) mental health history; iii) current level of support; iv) current drug or alcohol use; and v) past or current experience of domestic violence or abuse. Respondents provided a yes/no answer for the provision of each domain of assessment throughout the antenatal and postnatal periods. These domains of psychosocial assessment were chosen to reflect those addressed in key Australian initiatives, including the 2011 Guidelines [4].

#### Maternity hospital sector (index birth)

Respondents were asked to indicate where they had given birth to the index child and variables were collapsed to form three categories: public (public hospital and birthing centre); private (private hospital and private patient at a public hospital); other (at home / other).

#### Sociodemographic characteristics

Respondents’ partner and employment status, parity, highest level of education attained, background language, state/territory of residence and whether the residence was located in an urban or non-urban region were collected as part of the survey.

### Ethics

Ethical approvals for the study were granted from the Human Research Ethics Committees of the University of Newcastle (Ref: H-2010-0031), University of Queensland (Ref: 20100000411) and University of New South Wales (Ref: 10412).

#### Statistical analysis

Characteristics of women who did and did not report receiving health promotion information and/or psychosocial assessment were first compared using cross tabulations and chi-square tests. Log-binomial regression models were used to calculate prevalence ratios with 95% confidence intervals. This method of analysis was chosen in order to minimise any potential inflation of the magnitude of associations, which can occur with the use of logistic regression when the outcome prevalence is greater than 10% [26]. Inclusion of variables into the multivariate model for each outcome was based on significant results (p < .10) from univariate analyses (data not shown). For analyses by state, New South Wales was chosen as the reference category because at the time of data collection it was the single jurisdiction with state-level Department of Health perinatal mental health policy and guidelines [7,27,28]. Due to the small age range of participants (32–37 years), models were not adjusted for age. All analyses were performed using SAS 9.2 [29].

## Results

Seventy-nine percent of women (N = 1835) returned completed questionnaires between January 2011 – July 2011. Of these, 24 respondents did not meet eligibility criteria (index child born prior to 1 August 2007) and were subsequently excluded from the study, as were seven women who were living overseas at the time of survey completion. Thus the final sample comprised 1804 women.

The socio-demographic characteristics of participants are outlined in Table 1. Our study group consisted of predominately English speaking (93.6%) and tertiary level educated women (59.6% university and 23.8% technical colleges). Mean age was 34.8 years (SD = 1.45 years, range = 32-37 years). Nearly all the women were partnered (96.2%), 78.1% were multiparous, and 70.7% were employed at the time of the index birth. The proportion of women residing in urban or non-urban areas was 58.3% and 41.2%, respectively. Maternity care for the index pregnancy and birth was provided in the private sector for more than half the sample (53.6%). The mean age of the index child at the time of survey completion was 22.7 months (range = 0-46 months; SD = 11.3 months).

**Table 1:** Socio-demographic characteristics of study participants, mothers of the same age in the general population in Australia, and ALSWH main survey 5 participants (1973–1978 cohort)

**Characteristic**	**Study participants**	**General population**	**ALSWH**
			1973-1978 Cohort*
	**%**	**%**	**%**
**Partner status**^§^			
Married/de facto	96.2	91.8	77.4
Single/separated/divorced/widowed	3.4	6.4	22.6
*Missing/not stated*	0.4	1.8	0.4
**Parity**^§^			
Primiparous	21.9	30.7	30.6
Multiparous	78.1	69.2	69.4
*Missing/not stated*	-	0.1	*-*
**Income management**			
Not difficult	58.8		59.4
Difficult	40.7	n.a.	40.1
*Missing/not stated*	0.4		0.4
**Highest level of education**^**#**^			
University degree	59.6	23.5	50.8
Yr 12/Trade/ diploma	35.4	49.7	39.6
Year 10 or below	4.3	20.5	7.4
*Missing/not stated*	0.6	6.3	2.2
**Area of residence**^**#**^			
Urban^1^	58.3	68.2	54.6
Non-urban^2^	41.2	31.8	41.2
*Missing/not stated*	0.5	0.1	4.2
**Background language**^**#**^			
English	93.6	75.6	92.8
Non-English speaking	5.7	24.1	6.2
*Missing/not stated*	0.8	0.3	1.0
**Employment status**^**#**^			
Employed	70.7	59.7	77.3
Not employed	28.8	39.5	22.7
*Missing/not stated*	0.5	0.8	
**Hospital sector (index child)**^§^			
Private	53.5	41.4	
Public^3^	43.6	57.8	n.a.
Other	1.7	0.9	
*Missing/not stated*	1.2	-	

Source of general population comparison data: ^§^AIHW (unpublished) 2008 National Perinatal Data Collection; ^**#**^ABS, Customised Report, 2012 (2006/2011 Census data); *ALSWH 1973–1978 Cohort at Survey 5 (2009, N = 8, 200, from which the sampling frame for this study was drawn) except Background Language which was assessed at Survey 1 (1996); ^1^Major cities; ^2^Inner/outer regional & remote/very remote; ^3^Includes birthing centres; n.a: not available.

All 1804 women were included in subsequent analyses relating to the *antenatal* period. In order to capture information about psychosocial assessment during the complete first year following birth, only women (N = 1442) whose youngest child was 12 months of age or older were included in the *postnatal* analyses.

### Proportion of women who reported receiving perinatal mental health information and psychosocial assessment during the perinatal period

Overall rates of reported provision of perinatal mental health information and receipt of psychosocial assessment during pregnancy and the first postnatal year are presented in Table 2. At a national level, results indicate that there has been significant penetration of psychosocial assessment during the perinatal period, however some discrepancies exist between the domains of psychosocial health. A large majority of women reported receiving perinatal mental health information at least once during pregnancy (78.3%) and/or in the 12 months following birth (81.6%). Two-thirds of women (66.8%) reported being asked about their current emotional health in the antenatal period, increasing to 75.6% of women in the postnatal period. Overall, 52.9% of participants reported being asked about their mental health history at least once during pregnancy, and 41.2% at least once in the first postnatal year. Two-thirds of women (67.6%) were asked about drug or alcohol use during pregnancy, compared to approximately one third (35.3%) during the post natal period. Around 70% of women reported being asked about their level of support both before and after birth. Across the full perinatal period, women were *least* likely to be asked about their experience of domestic violence or abuse (35.5% during pregnancy; 31.8% in the postnatal period).

**Table 2:** Proportion of women who reported receiving perinatal mental health information and psychosocial assessment during the perinatal period, by State/Territory

**Outcome**	**Total**	**NSW**	**VIC**	**QLD**	**SA**	**WA**	**TAS**	**ACT**	**NT**
	**N (%)**	**N (%)**	**N (%)**	**N (%)**	**N (%)**	**N (%)**	**N (%)**	**N (%)**	**N (%)**
**During pregnancy**	(N = 1804)	(N = 494)	(N = 501)	(N = 373)	(N = 143)	(N = 163)	(N = 56)	(N = 48)	(N = 16)
Provision of PMH information	1412 (78.3)	391 (79.1)	372 (74.3)	289 (77.5)	112 (78.3)	132 (81.0)	49 (87.5)	46 (95.8)*	13 (81.2)*
*Psychosocial assessment domain*									
Current mental health	1205 (66.8)	350 (70.9)	287 (57.3)	240 (64.3)	95 (66.4)	131 (80.4)	40 (71.4)	41 (85.4)	12 (75.0)*
Mental health history	954 (52.9)	315 (63.8)	217 (43.3)	193 (51.7)	74 (51.7)	82 (50.3)	29 (51.8)	31 (64.6)	8 (50.0)
Level of support	1261 (69.9)	374 (75.7)	332 (66.3)	258 (69.2)	103 (72.0)	103 (63.2)	37 (66.1)	36 (75.0)	11 (68.8)
Drug and alcohol use	1220 (67.6)	355 (71.9)	308 (61.5)	258(69.2)	99 (69.2)	98 (60.1)	40 (71.4)	42 (87.5)	13 (81.2)*
Domestic violence or abuse	644 (35.7)	261 (52.8)	110 (22.0)	150 (40.2)	54 (37.8)	33 (20.2)	12 (21.4)	12 (25.0)	7 (43.8)
**During postnatal period**^**§**^	(N = 1442)	(N = 398)	(N = 394)	(N = 305)	(N = 114)	(N = 129)	(N = 44)	(N = 38)	(N = 14)
Provision of PMH information	1176 (81.6)	322 (80.9)	350 (88.8)	210 (68.9)	89 (78.1)	118 (91.5)	38 (86.4)	32 (84.2)	12 (85.7)*
*Psychosocial assessment domain*									
Current mental health	1090 (75.6)	303 (76.1)	331 (84.0)	179 (58.7)	75 (65.8)	115 (89.1)	39 (88.6)	30 (78.9)	12 (85.7)
Mental health history	594 (41.2)	190 (47.7)	144 (36.5)	84 (27.5)	58 (50.9)	64 (49.6)	23 (52.3)	20 (52.6)	8 (57.1)
Level of support	1011 (70.1)	291 (73.1)	298 (75.6)	159 (52.1)	85 (74.6)	100 (77.5)	34 (77.3)	28 (73.7)	11 (78.6)
Drug and alcohol use	509 (35.3)	184 (46.2)	115 (29.2)	73 (23.9)	52 (45.6)	42 (32.6)	19 (43.2)	13 (34.2)	8 (57.1)
Domestic violence or abuse	458 (31.8)	185 (46.5)	110 (27.9)	58 (19.0)	48 (42.1)	27 (20.9)	11 (25.0)	12 (31.6)	4 (28.6)**

*NSW* New South Wales, *VIC* Victoria, *QLD* Queensland, *SA* South Australia, *WA* Western Australia, *TAS* Tasmania, *ACT* Australian Capital Territory, *NT* Northern Territory, *PMH* Perinatal Mental Health, ^§^postnatal period defined as 0–12 months following birth; *N < 5 women *not* provided with perinatal mental health information or not assessed in that domain; **N < 5 women assessed in that domain.

#### Factors associated with psychosocial assessment across the perinatal period

Prevalence estimates by factors associated with psychosocial assessment across the perinatal period are detailed in Table 3. Table 4 shows the adjusted log-binomial regression models for factors associated with each domain of psychosocial assessment during pregnancy and the postnatal period.

**Table 3:** Prevalence estimates by factors associated with psychosocial assessment during the perinatal period

	**Given PMH information**	**Current emotional health**	**Mental health history**	**Level of support**	**Drug or alcohol use**	**Domestic violence or abuse**
	**N (%)**	**N (%)**	**N (%)**	**N (%)**	**N (%)**	**N (%)**
**Pregnancy (N = 1804)**						
**Partner status**						
Married/de facto	1357 (78.2)	1155 (66.6)	910 (52.4)	1207 (69.6)	1174 (67.7)	617 (35.6)
Single^1^	50 (80.6)	45 (72.6)	40 (64.5)	49 (79.0)	42 (67.7)	25 (40.3)
**Parity**						
Primiparous	327 (82.8)	258 (65.3)	224 (56.7)	268 (67.8)	292 (73.9)	137 (34.7)
Multiparous	1085 (77.0)	947 (67.2)	730 (51.8)	993 (70.5)	928 (65.9)	507 (36)
**Income management**^**2**^						
Not difficult	594 (80.8)	507 (69.0)	413 (56.2)	538 (73.2)	519 (70.6)	272 (37)
Difficult	812 (76.5)	692 (65.2)	537 (50.6)	718 (67.7)	697 (65.7)	370 (34.9)
**Highest level of education**						
University degree or higher	814 (75.7)	673 (62.5)	513 (47.7)	716 (66.5)	712 (66.2)	331 (30.8)
Year 12/Trade/ diploma	521 (81.4)	463 (72.3)	379 (59.2)	477 (74.5)	446 (69.7)	273 (42.7)
Year 10 or below	68 (88.3)	60 (77.9)	56 (72.7)	61 (79.2)	55 (71.4)	37 (48.1)
**Area of residence**^**3**^						
Urban	806 (76.6)	668 (63.5)	521 (49.5)	688 (65.4)	672 (63.9)	320 (30.4)
Non-urban	599 (80.6)	528 (71.1)	428 (57.6)	565 (76.0)	540 (72.7)	317 (42.7)
**Background language**^**4**^						
English	1335 (79.1)	1140 (67.5)	908 (53.8)	1197 (70.9)	1159 (68.7)	616 (36.5)
Other	67 (65.7)	57 (55.9)	42 (41.2)	59 (57.8)	56 (54.9)	24 (23.5)
**Employment status**^**5**^						
Employed	1001 (78.4)	833 (65.3)	652 (51.1)	880 (69.0)	870 (68.2)	442 (34.6)
Not employed	405 (78.0)	366 (70.5)	297 (57.2)	376 (72.4)	343 (66.1)	200 (38.5)
**Maternity care sector**^**6**^						
Private	692 (71.7)	542 (56.2)	380 (39.4)	550 (57.0)	545 (56.5)	177 (18.3)
Public	680 (86.4)	622 (79)	539 (68.5)	666 (84.6)	630 (80.1)	443 (56.3)
**State/Territory of residence**^**7**^						
NSW	391 (79.1)	350 (70.9)	315 (63.8)	374 (75.7)	355 (71.9)	261 (52.8)
VIC	372 (74.3)	287 (57.3)	217 (43.3)	332 (66.3)	308 (61.5)	110 (22.0)
QLD	289 (77.5)	240 (64.3)	193 (51.7)	258 (69.2)	258 (69.2)	150 (40.2)
SA	112 (78.3)	95 (66.4)	74 (51.7)	103 (72.0)	99 (69.2)	54 (37.8)
WA	132 (81.0)	131 (80.4)	82 (50.3)	103 (63.2)	98 (60.1)	33 (20.2)
Other	108 (90.0)	93 (77.5)	68 (56.7)	84 (70.0)	95 (79.2)	31 (25.8)
**Postnatal period (N = 1442)**						
**Partner status**						
Married/de facto	1127 (81.7)	1043 (75.6)	567 (41.1)	960 (69.6)	488 (35.4)	443 (32.1)
Single^1^	47 (78.3)	45 (75.0)	26 (43.3)	49 (81.7)	21 (35.0)	15 (25.0)
**Parity**						
Primiparous	328 (87.7)	294 (78.6)	165 (44.1)	277 (74.1)	143 (38.2)	128 (34.2)
Multiparous	848 (79.4)	796 (74.5)	429 (40.2)	734 (68.7)	366 (34.3)	330 (30.9)
**Income management**^**2**^						
Not difficult	502 (82.8)	456 (75.2)	261 (43.1)	422 (69.6)	233 (38.4)	194 (32.0)
Difficult	671 (80.7)	630 (75.8)	332 (40.0)	587 (70.6)	276 (33.2)	264 (31.8)
**Highest level of education**						
University degree or higher	689 (82.2)	648 (77.3)	331 (39.5)	580 (69.2)	282 (33.7)	261 (31.1)
Year 12/Trade/ diploma	435 (81.2)	397 (74.1)	238 (44.4)	384 (71.6)	208 (38.8)	179 (33.4)
Year 10 or below	48 (78.7)	41 (67.2)	24 (39.3)	43 (70.5)	19 (31.1)	18 (29.5)
**Area of residence**^**3**^						
Urban	672 (82.3)	616 (75.4)	330 (40.4)	571 (69.9)	284 (34.8)	259 (31.7)
Non-urban	499 (80.6)	469 (75.8)	261 (42.2)	435 (70.3)	222 (35.9)	196 (31.7)
**Background language**^**4**^						
English	1105 (82.2)	1026 (76.3)	563 (41.9)	953 (70.9)	483 (35.9)	437 (32.5)
Other	65 (74.7)	58 (66.7)	28 (32.2)	54 (62.1)	24 (27.6)	20 (23.0)
**Employment status**^**5**^						
Employed	854 (82.7)	794 (76.9)	434 (42.0)	725 (70.2)	369 (35.7)	336 (32.5)
Not employed	316 (78.8)	291 (72.6)	156 (38.9)	281 (70.1)	135 (33.7)	119 (29.7)
**Maternity care sector**^**6**^						
Private	628 (82.7)	576 (75.9)	292 (38.5)	518 (68.2)	242 (31.9)	216 (28.5)
Public	520 (80.7)	487 (75.6)	286 (44.4)	466 (72.4)	253 (39.3)	231 (35.9)
**State/Territory of residence**^**7**^						
NSW	322 (80.9)	303 (76.1)	190 (47.7)	291 (73.1)	184 (46.2)	185 (46.5)
VIC	350 (88.8)	331 (84.0)	144 (36.5)	298 (75.6)	115 (29.2)	110 (27.9)
QLD	210 (68.9)	179 (58.7)	84 (27.5)	159 (52.1)	73 (23.9)	58 (19.0)
SA	89 (78.1)	75 (65.8)	58 (50.9)	85 (74.6)	52 (45.6)	48 (42.1)
WA	118 (91.5)	115 (89.1)	64 (49.6)	100 (77.5)	42 (32.6)	27 (20.9)
Other	82 (85.4)	81 (84.4)	51 (53.1)	73 (76.0)	40 (41.7)	27 (28.1)

^1^ ‘Never married’, ‘single’, ‘widowed’, ‘divorced’, or ‘separated’; ^**2**^ Respondents were asked to indicate how they managed on their available income: item re-coded so that a zero score (‘not difficult’) included responses of ‘not too bad’ or ‘easy’, and a score of one (‘difficult’) indicated that income management was reported as ‘difficult some of the time’, ‘difficult all of the time’ or ‘impossible’; ^**3**^ Based on the Accessibility/Remoteness Index of Australia score., re-coded as ‘urban’ (major city) or ‘non-urban’ (regional or remote) [30]; ^4^ As reported at ALSWH 1973–1978 Cohort Survey 1 (1996); ^**5**^ Employment status at the time of the birth of the index child: item re-coded into two categories: ‘employed’ (full-time/part-time/casual) and ‘not employed’ (looking for work/not in paid workforce); ^**6**^ Public (public hospital and birthing centre); private (private hospital and private patient at a public hospital); ^7^*NSW* New South Wales, *VIC* Victoria, *QLD* Queensland, *SA* South Australia, *WA* Western Australia, Other: Tasmania, Australian Capital Territory, Northern Territory (collapsed into one category due to small cell sizes).

**Table 4:** Multivariate model for factors associated with psychosocial assessment during the perinatal period

	**Given PMH information**	**Current emotional health**	**Mental health history**	**Level of support**	**Drug or alcohol use**	**Domestic violence or abuse**
	**PR**^**§**^**[95% CI]**	**PR**^**§**^**[95% CI]**	**PR**^**§**^**[95% CI]**	**PR**^**§**^**[95% CI]**	**PR**^**§**^**[95% CI]**	**PR**^**§**^**[95% CI]**
**Pregnancy (N = 1804)**						
**Partner status**						
Married/de facto	**-**	**-**	1	1	**-**	**-**
Single^1^	**-**	**-**	0.89 [0.73 , 1.03]	1.04 [0.92 , 1.09]	**-**	**-**
**Parity**						
Primiparous	**1**	**-**	**1**		**1**	**-**
Multiparous	0.94 [0.90 , 0.99]*	**-**	0.87 [0.80 , 0.95]*		0.91 [0.86 , 0.96]**	**-**
**Income management**^**2**^						
Not difficult	**1**	1	1	1	**1**	**-**
Difficult	1.04 [1.00 , 1.09]*	1.02 [0.96 , 1.07]	1.04 [0.96 , 1.13]	1.03 [0.99 , 1.09]	1.06 [1.01 , 1.12]*	**-**
**Highest level of education**						
University degree or higher	-	1	**1**	1	**-**	1
Year 12/Trade/ diploma	-	1.06 [0.99 , 1.13]	1.08 [0.99 , 1.17]	1.04 [0.99 , 1.09]	**-**	1.07 [0.97 , 1.18]
Year 10 or below	-	1.05 [0.92 , 1.14]	1.24 [1.09 , 1.38]**	1.04 [0.93 , 1.13]	**-**	1.12 [0.89 , 1.31]
**Area of residence**^**3**^						
Urban	-	**1**	1	1	1	1
Non-urban	-	0.94 [0.90 , 1.00]*	0.96 [0.89 , 1.04]	1.03 [0.99 , 1.09]	0.99 [0.94 , 1.05]	1.03 [0.93 , 1.14]
**Background language**^**4**^						
English	1	1	1		1	1
Other	0.89 [0.77 , 1.01]	0.89 [0.74 , 1.01]	0.87 [0.68 , 1.03]		0.86 [0.71 , 1.00]	0.79 [0.56 , 1.04]
**Employment status**^**5**^						
Employed	**-**	1	1		**-**	**-**
Not employed	**-**	1.00 [0.94 , 1.06]	1.03 [0.95 , 1.12]		**-**	**-**
**Maternity care sector**^**6**^						
Private	**1**	**1**	**1**	**1**	**1**	**1**
Public	1.19 [1.13 , 1.25]**	1.41 [1.31 , 1.51]**	1.72 [1.57 , 1.89]**	1.45 [1.36 , 1.55]**	1.39 [1.30 , 1.49]**	2.96 [2.56 , 3.45]**
**#State/Territory of residence**^**7**^						
NSW	**1**	**1**	**1**	**1**	**1**	**1**
VIC	0.95 [0.89 , 1.02]	**0.81 [0.74 , 0.88]**	**0.71 [0.63 , 0.79]**	**0.89 [0.83 , 0.95]**	**0.89 [0.82 , 0.96]**	**0.45 [0.38 , 0.53]**
QLD	1.01 [0.94 , 1.07]	0.95 [0.87 , 1.04]	**0.86 [0.77 , 0.95]**	**0.94 [0.87 , 0.99]**	1.00 [0.92 , 1.06]	**0.85 [0.75 , 0.95]**
SA	1.00 [0.91 , 1.09]	0.92 [0.81 , 1.02]	**0.81 [0.69 , 0.93]**	0.94 [0.85 , 1.02]	0.94 [0.83 , 1.03]	**0.68 [0.55 , 0.82]**
WA	1.05 [0.96 , 1.12]	**1.12 [1.03 , 1.20]**	**0.82 [0.70 , 0.94]**	**0.85 [0.75 , 0.94]**	**0.87 [0.76 , 0.97]**	**0.42 [0.31 , 0.56]**
Other	**1.08 [1.01 , 1.14]**	**1.12 [1.00 , 1.20]**	0.91 [0.76 , 1.06]	**0.87 [0.76 , 0.96]**	1.08 [0.99 , 1.14]	**0.48 [0.34 , 0.63]**
**Postnatal period (N = 1442)**						
**Partner status**						
Married/de facto	**-**	**-**	**-**	1	**-**	**-**
Single^1^	**-**	**-**	**-**	1.10 [0.94 , 1.22]	**-**	**-**
**Parity**						
Primiparous	**1**	1	**-**	**1**	**-**	**-**
Multiparous	0.92 [0.88 , 0.96]**	0.95 [0.90 , 1.01]	**-**	0.93 [0.87 , 1.00]*	**-**	**-**
**Income management**^**2**^						
Not difficult	-	**-**	**-**	**-**	1	**-**
Difficult	-	**-**	**-**	**-**	1.15 [1.00 , 1.31]	**-**
**Background language**^**4**^						
English	-	**1**	1	1	**-**	**1**
Other	-	0.85 [0.72 , 0.97]*	0.78 [0.56 , 1.03]	0.83 [0.69 , 0.96]*	**-**	0.67 [0.44 , 0.95]*
**Maternity care sector**^**6**^						
Private	-	**-**	1	1	1	1
Public	-	**-**	1.09 [0.96 , 1.23]	1.01 [0.94 , 1.08]	1.14 [0.99 , 1.31]	1.11 [0.96 , 1.29]
^**#**^**State/Territory of residence**^**7**^						
NSW	**1**	**1**	**1**	**1**	**1**	**1**
VIC	**1.10 [1.04 , 1.17]**	**1.09 [1.02 , 1.17]**	**0.78 [0.66 , 0.92]**	1.02 [0.94 , 1.11]	**0.65 [0.54 , 0.79]**	**0.62 [0.51 , 0.74]**
QLD	**0.86 [0.78 , 0.94]**	**0.76 [0.68 , 0.85]**	**0.58 [0.47 , 0.72]**	**0.70 [0.62 , 0.79]**	**0.53 [0.42 , 0.66]**	**0.40 [0.31 , 0.52]**
SA	0.98 [0.87 , 1.08]	**0.85 [0.73 , 0.97]**	1.06 [0.85 , 1.30]	1.01 [0.88 , 1.13]	0.98 [0.77 , 1.22]	0.89 [0.69 , 1.12]
WA	**1.13 [1.06 , 1.21]**	**1.15 [1.06 , 1.24]**	1.04 [0.84 , 1.27]	1.04 [0.93 , 1.16]	**0.71 [0.54 , 0.92]**	**0.45 [0.31 , 0.63]**
Other	1.05 [0.95 , 1.15]	1.10 [0.98 , 1.20]	1.08 [0.85 , 1.33]	1.02 [0.88 , 1.15]	0.88 [0.66 , 1.13]	**0.57 [0.39 , 0.79]**

^*^p < 0.05; ^**^p < 0.001; ^**§**^PR: prevalence ratio; Inclusion of variables into the multivariate model for each outcome was based on significant results (p < .10) from univariate analyses; ^#^State was significant in all models at p < .001, except first model (pregnancy only) where significant at p = 0.0493. ^1^ ‘Never married’, ‘single’, ‘widowed’, ‘divorced’, or ‘separated’; ^**2**^ Respondents were asked to indicate how they managed on their available income: item re-coded so that a zero score (‘not difficult’) included responses of ‘not too bad’ or ‘easy’, and a score of one (‘difficult’) indicated that income management was reported as ‘difficult some of the time’, ‘difficult all of the time’ or ‘impossible’; ^3^ Based on the Accessibility/Remoteness Index of Australia score., re-coded as ‘urban’ (major city) or ‘non-urban’ (regional or remote) [30]; ^**4**^ As reported at ALSWH 1973–1978 Cohort Survey 1 (1996); ^5^ Employment status at the time of the birth of the index child: item re-coded into two categories:‘employed’ (full-time/part-time/casual) and ‘not employed’ (looking for work/not in paid workforce); ^6^ Public (public hospital and birthing centre); private (private hospital and private patient at a public hospital); ^7^ NSW New South Wales, VIC Victoria, QLD Queensland, SA South Australia, WA Western Australia, Other: Tasmania, Australian Capital Territory, Northern Territory (collapsed into one category due to small cell sizes).

### During pregnancy

During pregnancy, women who gave birth in the public sector were more likely to be assessed across *all* psychosocial domains than women who gave birth in the private sector, after controlling for other factors. Specifically, women receiving care in the public hospital system were approximately 40% more likely to be asked about their current emotional health (adjPR = 1.41, 95% CI: 1.31-1.51, p < 0.001), 45% more likely to be asked about their level of support (adjPR = 1.45, 95% CI: 1.36-1.55, p < 0.001), 72% more likely to be asked about their mental health history (adjPR = 1.72, 95% CI: 1.57-1.89, p < 0.001) and nearly three-times as likely to be asked about their experience of domestic violence or abuse (adjPR = 2.96, 95% CI: 2.56-3.45, p < 0.001), compared to private maternity patients.

State/territory of residence was also significantly associated with all domains of assessment in the antenatal period. The greatest differences between jurisdictions was in reported assessment of mental health history and experience of domestic violence or abuse, with prevalence estimates across states ranging from 43.3% to 63.8%, and 20.2% to 52.8%, respectively. Multiparous women were less likely than primiparous women to receive perinatal mental health information (adjPR = 0.94, 95% CI: 0.90-0.99, p = 0.010), or be asked about their mental health history (adjPR = 0.87, 95% CI: 0.80-0.95, p = 0.002) or drug and alcohol use (adjPR = 0.91, 95% CI: 0.86-0.96, p < 0.001), in the antenatal period. Women with a lower educational level were more likely to be asked about mental health history than women with higher educational levels (adjPR = 1.24, 95% CI: 1.09-1.38, p < 0.001), however educational status was not significantly associated with any other domain of assessment. Area of residence was significantly associated with assessment of current emotional health, with women residing in non-urban areas just 6% less likely to be asked this than women in urban areas (adjPR = 0.94, 95% CI: 0.90-1.00, p < 0.001), but was not associated with any other domain of assessment. Partner status, background language and employment status were not significantly associated with any domain of psychosocial assessment during pregnancy, after controlling for other factors.

### Postnatal period

State/territory of residence was significantly associated with all domains of assessment in the postnatal period, with different states faring better in different domains. For example, prevalence estimates for receipt of perinatal mental health information and assessment of current mental health were greatest among women who resided in Western Australia (91.5% and 89.1%, respectively), whereas NSW-based women were up to 60% more likely to be asked about domestic violence or abuse than women residing in all remaining states/territories, except South Australia. Women residing in Queensland were less likely to be assessed across all psychosocial domains, when compared to NSW residents.

Multiparous women were less likely than primiparous women to receive perinatal mental health information (adjPR = 0.92, 95% CI: 0.88-0.96, p < 0.001), or be asked about their level of support (adjPR = 0.93, 95% CI: 0.0.87-1.00, p = 0.049). Women with a background language other than English were 15% less likely to be asked about their current emotional health (adjPR = 0.85, 95% CI: 0.72-0.97, p = 0.029), 17% less likely to be asked about their level of support (adjPR = 0.83, 95% CI: 0.69-0.96, p = 0.025) and 33% less likely to be asked about their experience of domestic violence or abuse (adjPR = 0.67, 95% CI: 0.44-0.95, p = 0.043), compared with women whose first language was English.

Maternity care sector, partner status, income management, educational level, area of residence and employment status and hospital maternity sector were not significantly associated with receipt of any domain of psychosocial assessment in the postnatal period, after controlling for other factors.

## Discussion

This is the first Australian study that has investigated factors that influence equity of access to assessment programs across the perinatal period. Our results indicate that there has been significant penetration of some components of psychosocial assessment in Australia both in antenatal and postnatal periods, including assessment of current mental health. Disappointingly, however, rates decreased markedly for reported assessment of mental health history – one the most predictive factors for perinatal relapse [31-34] – and for assessment of domestic violence or abuse, despite experience of violence and abuse being associated with serious health consequences, including postnatal depression [35]. These findings suggest that although investigation of current emotional health has been broadly embraced, much less examination is occurring in terms of more in depth assessment of a women’s psychosocial health during the perinatal period. This study also shows that maternity hospital sector was significantly associated with receipt of all domains of assessment during pregnancy, although this was not the case in the postnatal period.

These findings are particularly significant when viewed in the context of the delivery of maternity services across the public and private sectors in Australia, existing state and national guidelines relating to maternity care, and availability of supporting resources. First, approximately a third of all women give birth in private maternity settings in Australia [36] yet this study indicates that these women are at a particular disadvantage in terms of reported participation in perinatal psychosocial assessment programs during pregnancy. It may be argued that this finding reflects effective implementation of psychosocial assessment, insofar as the least advantaged group who may have the highest needs and risks are being assessed. However this view minimises the very real risk factors and psychological morbidity that can be experienced by women of all socioeconomic, employment and educational backgrounds [37,38], and is counter to the *universal* approach to perinatal psychosocial assessment that is advocated in the Australian setting. That this disparity among public and private patients did not extend to the postnatal period is less surprising, given that the provision of maternity care in the private sector in Australia is generally confined to pregnancy, delivery and the immediate postpartum period.

Encouragingly, isolated examples of perinatal psychosocial assessment programs are emerging in private maternity hospitals (e.g., [39]), and it will be important to monitor the extent to which women whose maternity care is provided these settings are included in perinatal prevention and early intervention initiatives. This is relevant not only because the 2011 Guidelines are unequivocally inclusive of the private sector, but also because the universal, routine approach to perinatal psychosocial assessment recommended in these Guidelines has been endorsed by the Royal Australian and New Zealand College of Obstetricians and Gynaecologists [40]. However, broader and more effective implementation of assessment in this sector will require a deeper understanding of why some of the most socio-economically advantaged women are least likely to be asked about their psychosocial health. Factors which underpin this finding may include that that clinicians providing antenatal care in the private sector adhere to the stereotype that affluent women do not experience perinatal depression or anxiety [41], that women do not see their obstetrician as being the appropriate person to consult about their symptoms [42], that time pressures in a busy clinical setting results in a reactive rather than proactive approach to assessment and management of perinatal mental health issues [41], or a combination of these.

Second, although the 2011 Guidelines were released simultaneously with the period of data collection for this study (and so could not be expected to have had an influence on the uptake of psychosocial assessment reported here), a number of guidelines which are inclusive of at least some components of perinatal psychosocial assessment have been in place at local, state or health provider levels for a number of years (e.g., [7,8,43,44]), and may account for some of the differences identified across domains and across States/Territories. In addition, the *beyondblue* Postnatal Depression Program conducted feasibility studies of ante- and postnatal depression screening across Australia, delivered in tandem with training for health professionals and a widespread community awareness campaign, between 2001 and 2005 [1]. This program may have resulted in State-based and local initiatives and practices that continued after the feasibility study was completed and as the National Action Plan for Perinatal Mental Health was developed and promoted [14].

Taking as a second example screening for domestic violence: a number of state health departments in Australia recommend universal routine screening for domestic violence for women attending antenatal and/or early childhood health services [45-49]. These recommendations are mirrored by e.g., the Royal Australian College of General Practitioners, which has recommended opportunistic screening for intimate partner violence for all women who are pregnant since 2009 [43], and in principle should capture the 15% of women whose antenatal care is provided in the general practice setting. However, despite the implementation of these policies and guidelines, only about one third of women in this study reported being asked about their experience of violence or abuse during pregnancy (35.7%) or in the year following birth (31.8%), significantly less than the proportion of women who reported being asked about their current emotional health (66.8% and 75.6% during pregnancy and the postnatal period, respectively). The proportion of women reporting assessment of violence or abuse across the *full* perinatal period was significantly greater in New South Wales, where domestic violence screening has been progressively introduced in antenatal and early childhood services since 2001, and thus may reflect the earlier implementation of policies relating to screening for domestic violence in that state, relative to other states/territories. However, it was not possible in this study to examine this or differences among states in additional domains of assessment in more detail due to the low numbers of respondents in some jurisdictions, and due to the implementation of a range of state-based initiatives relative to the timing of our data collection.

It is also not possible to ascertain from this study if variations in reported rates of assessment across each of the psychosocial domains reflects resource issues and/or attitudinal issues on the part of the health professional or consumer, or both. Previous research has highlighted the importance of adequate training and resources, structured referral pathways for women and supportive systems for staff in supporting the successful implementation of psychosocial assessment programs [50-55], particularly when assessing women with complex psychosocial presentations, including the presence of domestic violence [54]. In addition, it has been suggested that the increasing focus on perinatal *depression* may result in neglect of other aspects of psychosocial wellbeing in the perinatal period [17].

Third, this study shows women with a background language other than English are less likely to be asked about their current emotional health, level of support or experience of violence or abuse in the postnatal period. While it is encouraging that these differences were evident in only a limited of number of domains, that such disparities remain is concerning given the availability of locally-produced resources which aim to facilitate the delivery of these components of psychosocial assessment to culturally and linguistically diverse families. Such resources include, but are not limited to, translated versions of the EPDS [56] which should be utilised to meet the needs of ethnically diverse communities of women who give birth in Australia [36]. Of note is that background language was not associated with receipt of psychoeducational material during pregnancy or following birth, and may reflect the widespread availability and dissemination of resources such as the *beyondblue* ‘Emotional Health During Pregnancy & Early Parenthood’ information booklet (freely available in over 20 languages) [57]. That multiparous women were less likely to be given information or be asked about a number of domains of psychosocial health, including past mental health, is also concerning, particularly in light of the international recognition given to previous pregnancy or postnatal mental health episodes as a specific risk factor for poorer perinatal mental health outcomes (e.g., [4,58,59].

These data need to be viewed in the context of the study’s limitations and strengths. In comparison to mothers of the same age in the general population in Australia [60,61], our sample had higher proportions of partnered, multiparous, employed and tertiary-level educated women, and a higher proportion of women who gave birth in the private maternity sector While this limits the generalisability of our results, the inclusion of the latter group of women in our sample provided a unique opportunity to examine reported access to perinatal psychosocial assessment in the private maternity sector relative to the public maternity sector. Given that some 30% of births in Australia occur in private hospitals, it is imperative that outcomes for these women are not ignored in perinatal mental health research.

In addition, the age range of respondents was limited to 32–37 years, and thus was not representative of all women of reproductive age. Younger age has been reported to be a risk factor for poorer perinatal mental health outcomes (e.g., [5,18]), although other studies have showed contrary findings (e.g., [16,62]). Regardless, psychosocial assessment (including depression screening) is recommended as a routine component of care for *all* women who are pregnant or have recently given birth in Australia [4]. It follows then that examination of the extent of participation in these programs should apply to any given cross-section of the perinatal population, and should not be limited to sub-groups considered particularly vulnerable or at risk.

It is not possible to ascertain if reported assessment of current emotional health was by means of recommended tools (e.g., the EPDS), although existing research has shown that around 80% of women assessed during their pregnancy in the public maternity setting were given the EPDS [13]. The interpretation of our findings is also limited by the fact that receipt of psychosocial assessment during pregnancy and following birth was identified by means of self-report and, therefore, may be open to the effects of recall bias. Existing studies which have examined maternal recall of perinatal events have shown that accuracy and reliability of recall varies by topic (e.g., obstetric versus lifestyle events) [63,64]. However, these studies have not addressed recall of issues relating to emotional health, and the periods of recall they describe (10–15 years) are longer than that required our respondents. Thus it is difficult to determine the magnitude of bias (if any) associated with the average two-year recall period encountered in this study. We cannot discard the possibility that experiencing emotional distress during the perinatal period, for example, could have influenced a woman’s recall of whether or not she received psychosocial assessment however one would imagine that a woman who felt in need of additional support would remember whether she was asked by a health professional about her emotional health more clearly. Alternatively, we cannot discount that additional factors such as current mood state, or a particular interest in the key themes of the survey instrument, may have an impact on retrospective reporting. Recall bias is a concern for any study of this type, however the extent to which the rates of psychosocial assessment reported in this study are an under- or over- estimate of true clinical practice is unknown since in Australia information relating to perinatal depression screening and psychosocial assessment is not routinely collected in national administrative health datasets.

## Conclusions

Despite these limitations, the present study provides an important insight into the reported overall uptake of and access to perinatal psychosocial assessment among a large sample of women in Australia. The need to minimise the current shortfall in assessment rates in the private maternity setting, particularly during pregnancy, is especially evident and should be addressed as a priority. Endorsement of the recommendations and good practice points of the 2011 Clinical Practice Guidelines for Perinatal Mental Health by RANZCOG [40] and the implementation of routine assessment programs at selected private hospitals [39] are important first steps, however ongoing engagement with both clinicians and consumers in this important sector is required if the challenges of embracing a comprehensive and inclusive approach to perinatal psychosocial assessment (including targeted education and awareness campaigns, ensuring adequate workforce capacity, access to quality training programs, and identification of appropriate pathways to care) are to be met. Similarly, locally produced resources and initiatives which aim to bridge the gap in the delivery of perinatal mental health services to women from culturally and linguistically diverse backgrounds are freely available and should be utilised [56,57,65].

This results of this study are particularly important given that there is currently no capacity to monitor the extent of implementation of the NPDI or the 2011 Guidelines at a national level [66]. Without a nationally consistent approach to data development relating to perinatal mental health, it will remain difficult to ascertain if these key initiatives have had the desired effect of increasing rates of depression screening and psychosocial assessment among perinatal populations in service areas where implementation has occurred. The absence of this information is most significant when considered in light of the performance benchmarks established as part of the Implementation Plan for the National Perinatal Depression Initiative – National Partnership Agreement on Health Services [67]. Use of data from this sample of women will help to fill this gap, until such time as national data elements are developed and implemented.

## Competing interests

The authors declare that they have no competing interests.

## Authors’ contributions

NR conceived of the study, participated in its design and coordination, performed the statistical analysis and drafted the manuscript. SH performed the statistical analysis. DL, CC, PF, JM and MPA participated in the design, and DL and CC in the coordination, of the study. DL and PF advised on statistical issues. All authors read and revised the manuscript critically, and approved the final version.

## Authors’ information

Sheree Harris, Deborah Loxton, Catherine Chojenta, Peta Forder, Jeannette Milgrom and Marie-Paule Austin are co-authors.

## Pre-publication history

The pre-publication history for this paper can be accessed here:

http://www.biomedcentral.com/1471-2458/13/632/prepub
